# Human Papillomavirus Detection from Human Immunodeficiency Virus-Infected Colombian Women's Paired Urine and Cervical Samples

**DOI:** 10.1371/journal.pone.0056509

**Published:** 2013-02-13

**Authors:** Marina Munoz, Milena Camargo, Sara C. Soto-De Leon, Ricardo Sanchez, Diana Parra, Andrea C. Pineda, Otto Sussmann, Antonio Perez-Prados, Manuel E. Patarroyo, Manuel A. Patarroyo

**Affiliations:** 1 Fundación Instituto de Inmunología de Colombia (FIDIC), Bogotá, Colombia; 2 School of Medicine and Health Sciences, Universidad del Rosario, Bogotá, Colombia; 3 Universidad Nacional de Colombia, Bogotá, Colombia; 4 Asistencia Científica de Alta Complejidad S.A.S., Bogotá, Colombia; 5 Universidad Pública de Navarra, Pamplona, Spain; Vanderbilt University, United States of America

## Abstract

Infection, coinfection and type-specific human papillomavirus (HPV) distribution was evaluated in human immunodeficiency virus (HIV)-positive women from paired cervical and urine samples. Paired cervical and urine samples (n = 204) were taken from HIV-positive women for identifying HPV-DNA presence by using polymerase chain reaction (PCR) with three generic primer sets (GP5+/6+, MY09/11 and pU1M/2R). HPV-positive samples were typed for six high-risk HPV (HR-HPV) (HPV-16, -18, -31, -33, -45 and -58) and two low-risk (LR-HPV) (HPV-6/11) types. Agreement between paired sample results and diagnostic performance was evaluated. HPV infection prevalence was 70.6% in cervical and 63.2% in urine samples. HPV-16 was the most prevalent HPV type in both types of sample (66.7% in cervical samples and 62.0% in urine) followed by HPV-31(47.2%) in cervical samples and HPV-58 (35.7%) in urine samples. There was 55.4% coinfection (infection by more than one type of HPV) in cervical samples and 40.2% in urine samples. Abnormal Papanicolau smears were observed in 25.3% of the women, presenting significant association with HPV-DNA being identified in urine samples. There was poor agreement of cervical and urine sample results in generic and type-specific detection of HPV. Urine samples provided the best diagnosis when taking cytological findings as reference. In conclusion including urine samples could be a good strategy for ensuring adherence to screening programs aimed at reducing the impact of cervical cancer, since this sample is easy to obtain and showed good diagnostic performance.

## Introduction

The human immunodeficiency virus (HIV) is a sexually-transmitted infection (STD) having a great impact around the world due to the large amount of people living with such infection (34.2 million) and the frequent appearance of new cases (2.5 million in 2011) [1]. It is characterized by affecting immune system CD4+ cells, thereby leading to a reduction in the body's efficiency regarding the presentation of a response against other pathogens, making an individual more vulnerable to other types of infection [2].

Some studies have suggested that women living with HIV/AIDS have increased frequency and incidence of single and multiple infections caused by human papillomavirus (HPV) [3]; the natural history of infection becomes altered, thereby leading to an increased risk of developing cervical cancer (CC) and contributing towards this type of cancer being the most frequently diagnosed in HIV-positive women [4]. This relationship may be due to: higher HPV exposure in HIV-infected women, increased frequency of main risk factors involved in CC development or the role of HIV-related immunosuppression in favoring carcinogenesis [5].

The immunosuppression can be attenuated through using antiretroviral therapy which favors balanced counts of CD4 lymphocytes, however, this therapy has not been consistently implicated in the reduction of HPV-related diseases [6].

The CC incidence in the Colombian general population is 36.4 cases/year/100,000 women [7]; the disease onset occurs approximately between 7 and 12 years after initial HPV infection [8]. These clinical features are altered in women infected simultaneously with HPV and HIV where a short-term clinical outcome usually occurs, involving lesions developing more aggressively, slower HPV infection regression rates and poorer responses to treatment [9]; such factors mean that pre-cancerous lesions could reach 60% (evolving in less than 3 years) [10].

Cervical cytology is the most widely used strategy for reducing the cervical cancer burden around the world [11]. However, this screening test has reduced impact in HIV-infected women, as this group has a greater probability of becoming infected with HPV and developing cervical lesions [12], which has led to cytological screening guidelines being rewritten, now including a test every six months during the first year followed by a yearly check-up scheme if no lesions are observed [13]. Nevertheless, cytology coverage in this group of women is poor and insufficient [10], therefore, monitoring programs that allow the constant screening in extended time periods is thus suggested, considering the high risk associated with this group of women.

In view of the above, the use of complementary techniques to the Papanicolau test could represent a useful tool in detecting women at risk. Some of these methods are non-invasive, such as self-sampling, as when they are used in screening programs they could provide advantages related to increased acceptance regarding sample-taking, adherence and following-up women, especially those having some form of immunological compromise [14], [15].

Specimen tampons, vaginal swabs and urine samples have been studied as self-sampling methods; such sampling methods are also used for detecting other sexually-transmitted pathogens affecting the cervical area [9], [16], urine samples being the easiest to obtain and having had the greatest acceptance in the population. However, they do have some limitations, including low cellular load and they are not taken directly from the HPV infection site; this could mean that the results obtained from this type of sample might not reflect the real clinical state of an infection [14].

In spite of their limitations, using urine samples as a test for detecting HPV-DNA presence could facilitate frequent sample-taking due to their practicality and greater acceptance among women. This could be useful in studies involving a large number of samples and a pelvic examination is also not required, meaning that sample-taking will not affect the natural history of HPV infection as there is no risk of micro-lesions being produced, nor will inflammatory reactions occur [15].

Despite of multiple studies available in the literature that have evaluated HPV-DNA detection from urine sample [15], a few number of these have been described the diagnostic performance of this sample in HIV-positive women population. Furthermore those who have done it had included a limited number of individuals [9], [17].

In Colombia high prevalence of HPV infection and co-infection in healthy women population have been reported, using cervical samples [18], [19]. However haven't be evaluated HPV DNA detection from urine samples neither in HIV-positive women population.

This study aimed at identifying the infection, coinfection (defined here as being infection by more than one type of HPV simultaneously) and type-specific distribution profile of six high-risk HPV (HR-HPV) types and two low-risk (LR-HPV) types, from paired cervical and urine samples of women diagnosed with HIV/AIDS, confirmed by Western blot. Finally, we evaluated the diagnostic performance of urine samples compared to cervical samples for detecting HPV infection.

## Materials and Methods

### Study population and sample size

HIV-infected women (such infection having been confirmed by Western blot) participating in cervical cancer screening campaigns being offered by the Centro de Asistencia Científica de Alta Complejidad S.A.S., in Bogotá, Colombia, were included in the present study. The study was approved and supervised by the participating institutions' ethics committees: Fundación Instituto de Inmunología de Colombia's ethics committee and Centro de Asistencia Científica de Alta Complejidad S.A.S.' ethics committee.

Sample size was calculated assuming an estimated 80% HPV infection rate in HIV-positive women [4], [17], [20], according to data reported in the literature. Estimators were calculated using 0.05 precision along with 95% confidence intervals (95%CI) using STATA9 software sampsi command.

### Collecting and processing cervical and urine samples

All the women enrolled in the study were informed about the research objective; they signed an informed consent form and filled in a questionnaire to facilitate collecting socio-demographic data and information regarding their sexual habits and other risk factors related to acquiring HPV infection.

Each woman's urine and cervical samples were taken on the same day; the first sample from a midstream urine specimen was self-collected, kept at 4°C and processed within 72 hours after being collected. The second sample taken from cervical cells was obtained during Papanicolau test, following Colombian obligatory health plan guidelines regarding cervical cancer detection and control programs in Colombia [21]; these cells were preserved in 95% ethanol [22], [23] and kept at 4°C until being processed. The histological findings were reported following the Bethesda classification [13].

The cells were precipitated by spinning at 2,300× *g* for 20 minutes at 4°C for urine samples and at 15,000× *g* for 10 minutes at 4°C for cervical samples. DNA was extracted from cell pellets of paired samples using a QuickExtract DNA extraction kit (Epicentre, Madison, WI), following the manufacturer's instructions. Two PCR amplifications were made with specific primers directed at a segment of the human β-globin constitutive gene (GH20/PC04 and PC03/PC04) for evaluating DNA integrity [18], [22], [24].

### Detecting human papillomavirus DNA by PCR amplification

Samples yielding a positive result for the human β-globin gene were amplified for detecting HPV using three consensus primer sets (for detecting more infected women) as it has been reported that using a single set might lead to underestimating viral prevalence compared to studies using more than one generic detection system [25].

Two of the primers sets were directed to the region encoding late viral protein L1: GP5+/6+ and MY09/11 [26], [27]; PCR conditions have been described previously [22]. A third set of primers (pU1M/2R) was directed to the HR-HPV E6/E7 region [28], [29]. Assays were run in a final 25 µl volume. The mix contained 1× amplification buffer, 100 µM of each dNTP, 2.5 mM MgCl2, 1 U MangoTaq DNA polymerase (Bioline, London, UK) and 1 µM of each primer. The following amplification profile was used: an initial denaturing step at 94°C for 10 min, followed by 30 amplification cycles lasting 1 min at 94°C, 2 min at 53°C and 2 min at 72°C, followed by a final extension step for 7 min at 72°C.

Generic primer sets' HPV-DNA detection analytical sensitivity has been reported in previous studies, the detection limit being 10^2^ and 10^4^ plasmid copies in 100 ng HPV-DNA for HPV-16 and -45, respectively, using a GP5+/6+ primer set [30], 10 HPV copies of HPV-31 type and 10^2^ HPV copies of HPV-16, -18, -33, -45 and -58 using an MY09/11 primer set [31] and 0.1 copies of the HPV-16 genome per cell using a pU1M/2R primer set [28].

Samples which proved positive for any of the three generic primer sets were amplified by PCR for identifying the six HR-HPV viral types (HR-HPV-16, -18, -31, -33, -45, -58), using type-specific primers targeting the E5, E6 and E7 regions, according to that reported in the literature for each viral type [22], [32], [33]. Two types of LR-HPV were detected (LR-HPV-6/11) [22], [34]. The amplification products were visualized on 2% agarose gels for human β-globin and the three generic reactions, and on 2.5% gels for the type-specific reactions. All gels were stained with SYBR Safe (Invitrogen, Carlsbad, CA).

### Statistical analysis

Means and standard deviations were used for describing continuous variables; categorical variables were expressed in terms of frequencies and percentages. The frequency of events of interest was reported together with their corresponding 95% confidence intervals that were calculated using the bootstrap method. The association between categorical variables was evaluated with Chi-square (χ2) tests, using a significance level of 0.05.

Agreement between HPV results for urine and cervical samples was evaluated using the kappa coefficient (κ), and its corresponding 95%CI, classified as follows: negative values, as well as values from 0 to 0.2 = poor, from 0.21 to 0.4 = slight, from 0.41 to 0.6 = fair, from 0.61 to 0.8 = moderate, from 0.81 to 0.99 = substantial and 1.0 = perfect agreement [27]. The urine HPV tests' operative characteristics were evaluated for determining sensitivity, specificity, positive and negative predictive values and receiver operating characteristic (ROC) area (as the average of sensitivity and specificity), taking the HPV cervical sample result as reference. STATA 9 software was used for all statistical analysis.

## Results

### Socio-demographic data

Two hundred and forty five women between 20 and 73 years old, were enrolled in the study (mean age: 38.1 years; SD 10.7 years) (Table 1). Two hundred and thirty nine of the 245 cervical samples (97.6%) were positive by human β-globin amplification and 208 of the 226 urine samples (92.4%). Fifty one women were not included in the statistical analysis due to their samples' low DNA quality (negative result for β-globin) or a lack of either of the samples (cervical or urine).

**Table 1:** Description of the socio-demographic characteristics of the female population enrolled in the study.

Characteristic	Categories	n (%)*
Age, years (201)	20–34	91 (45.3)
	35–49	72 (35.8)
	≥50	38 (18.9)
Ethnicity (197)	White	132 (67.0)
	Indigenous	3 (1.5)
	Mestizo	58 (29.5)
	Black	4 (2.0)
Marital status (186)	Single	52 (27.9)
	Married	24 (12.9)
	Common law marriage	60 (32.3)
	Separated	21 (11.3)
	Widowed	29 (15.6)
Age at first intercourse (195)	<18	118 (60.5)
	≥18	77 (39.5)
Pregnancies (192)	None	9 (4.7)
	1	45 (23.4)
	2	51 (26.6)
	3	47 (24.5)
	≥4	40 (20.8)
Life time number of sexual partners (194)	1	30 (15.5)
	2	48 (24.7)
	3	43 (22.2)
	≥4	73 (37.6)
Contraceptive method** (165)	None	33 (20.0)
	Hormonal contraceptives	5 (3.0)
	Intrauterine device	11 (6.7)
	Surgery	35 (21.2)
	Condom	54 (32.7)
	Condom + other	27 (16.4)
Smoking status (193)	No	163 (84.5)
	Yes	30 (15.5)

* Categories have a size lower than 204, given that data was missing from the surveys.

** Contraceptive method used at the moment of enrollment in this study.

### Human papillomavirus prevalence and type-specific distribution

**Figure 1:**
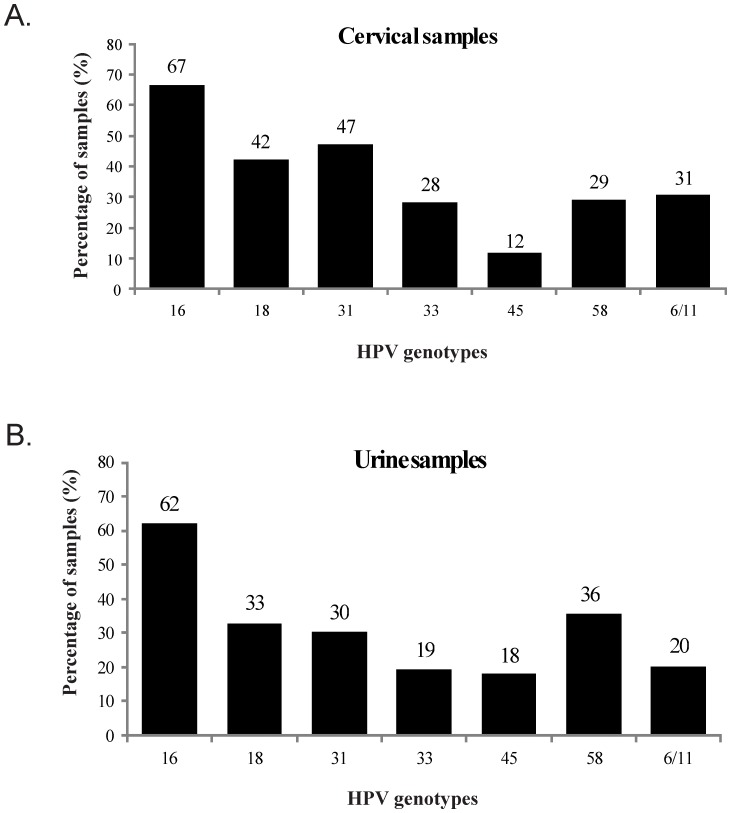
Prevalence of HPV types in cervical and urine samples for 204 HIV-infected Colombian women.

HPV infection frequency in cervical and urine samples was 70.6% (n = 144; 63.8–73.7 95%CI) and 63.2% (n = 129; 56.2–69.9 95%CI), respectively. Type-specific viral identification revealed that HPV-16 had the greatest prevalence in both samples, whilst HPV-31 had the second greatest prevalence in the cervical samples and HPV-58 in urine samples; the other viral types had a variable distribution in both samples (Figure 1).

**Figure 2:**
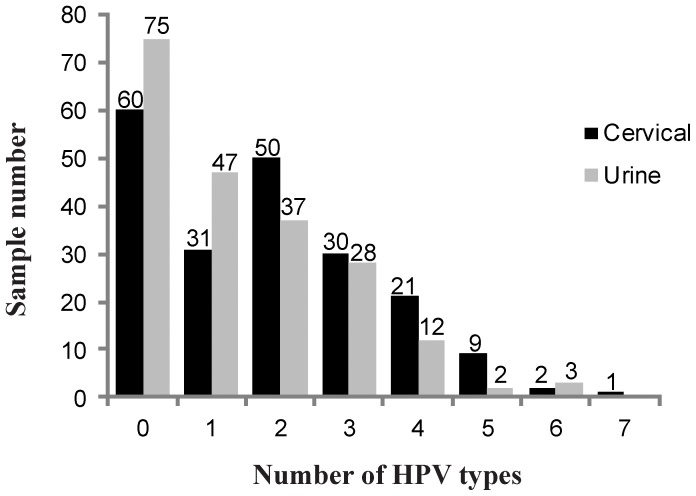
Number of HPV types in cervical and urine samples for 204 HIV-infected Colombian women.

It was found that 55.4% (n = 113; 95% CI = 48.3–62.3) of the cervical samples had coinfection, compared to 40.2% (n = 82; 33.4–47.3 95%CI) of the urine samples. Regarding a description of the number of types of HPV simultaneously present in each sample analyzed, urine samples revealed more uninfected women or those having just one HPV-type compared to the results obtained for cervical samples where more coinfections were detected (2 to 8 types of HPV). The presence of multiple infection per sample type had a statistically significant relationship (Fisher's exact test, p = 0.000) (Figure 2).

### Cytological abnormalities and HPV presence

The Papanicolau test gave abnormal cytological findings in 28.9% of the population (n = 56; 95% CI = 22.6–35.8), results being classified as follows: 10.3% (n = 20) had atypical squamous cells of undetermined significance (AS-CUS), 16.5% (n = 32) low-grade squamous intraepithelial lesions (L-SIL) and 2.1% (n = 4) high-grade squamous intraepithelial lesions (H-SIL).

The HPV infection results obtained from the two samples were classified according the cytological results; data are shown in Table 2.

**Table 2:** HPV detection in both sample sources according to the cytological results.

	HPV detection n (%)				
	Both positive	Cytology only	Urine only	Both negative	Total (194)
Normal	57 (41.3)	35 (25.4)	22 (15.9)	24 (17.4)	138
ASC-US	12 (60.0)	4 (20.0)	3 (15.0)	1 (5.0)	20
L-SIL	24 (75.0)	1 (3.1)	3 (9.4)	4 (12.5)	32
H-SIL	2 (50.0)	1 (25.0)	1 (25.0)	0 (0.0)	4

The percentages show the frequency of women that tested positive with respect to the total per row. **AS-CUS**: Atypical squamous cells of undetermined significance. **H-SIL**: high-grade squamous intraepithelial lesions. **L-SIL**: Low-grade squamous intraepithelial lesions.

The association between the presence of HPV-DNA in each sample and the cytological findings (categorized as being normal/abnormal) revealed that 20.4% (n = 12) of the women having abnormal cytological findings had a negative result for HPV infection in the cervical sample while 78.6% gave a positive result (n = 44). Such difference was not statistically significant (χ^2^(1) = 2.69; p = 0.101). On the other hand, it was found that 19.6% (n = 11) of the samples having abnormal cytological findings had negative test for HPV-DNA in the urine sample, compared to 80.4% (n = 45) where viral DNA was detected; this trend was statistically significant (χ^2^(1) = 9.22; p = 0.002).

### Agreement between both samples and clinical performance

The results obtained for infection (generic and type-specific) in both samples (cervical and urine) were compared to cytological findings, categorizing the population as normal or abnormal (Tables 3-A and 3-B, respectively), where the generic identification of HPV-DNA, showed the greatest percentage agreement.

**Table 3:** HPV detection and type-specific distribution from each source sample (cervical and urine) in the group of women having normal and abnormal cytological findings.

	Women having a normal cytology result (n = 138)																Women having an abnormal cytology result (n = 56)															
	n (%)																n (%)															
	Both positive				Cervical sample only				Urine sample only				Both negative				Both positive				Cervical sample only				Urine sample only				Both negative			
HPV infection*	57	(	41.3	)	35	(	25.4	)	22	(	15.9	)	24	(	17.4	)	38	(	67.9	)	6	(	10.7	)	7	(	12.5	)	5	(	8.9	)
HPV-16	23	(	20.2	)	41	(	36.0	)	22	(	19.3	)	28	(	24.5	)	14	(	27.5	)	12	(	23.5	)	17	(	33.3	)	8	(	15.7	)
HPV-18	6	(	5.3	)	33	(	28.9	)	19	(	16.7	)	56	(	49.1	)	6	(	11.8	)	12	(	23.5	)	10	(	19.6	)	23	(	45.1	)
HPV-31	7	(	6.1	)	31	(	27.2	)	17	(	14.9	)	59	(	51.8	)	5	(	9.8	)	21	(	41.2	)	9	(	17.6	)	16	(	31.4	)
HPV-33	4	(	3.5	)	20	(	17.6	)	12	(	10.5	)	78	(	68.4	)	5	(	9.8	)	11	(	21.6	)	3	(	5.9	)	32	(	62.7	)
HPV-45	0	(	0.0	)	7	(	6.2	)	12	(	10.5	)	95	(	83.3	)	1	(	2.0	)	7	(	13.7	)	10	(	19.6	)	33	(	64.7	)
HPV-58	4	(	3.5	)	20	(	17.5	)	22	(	19.3	)	68	(	59.7	)	6	(	11.8	)	9	(	17.6	)	13	(	25.5	)	23	(	45.1	)
HPV-6/11	2	(	1.8	)	20	(	17.5	)	14	(	12.3	)	78	(	68.4	)	5	(	9.8	)	13	(	25.5	)	2	(	3.9	)	31	(	60.8	)

* The positivity percentage for HPV infection (using generic primers) in each sample source. Type-specific identification was used in some HPV infection-positive women regarding any of the sample sources (n = 114 and n = 51 for the groups of women having normal or abnormal cytology result, respectively).

**Figure 3:**
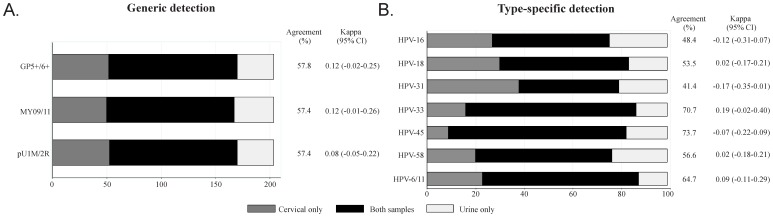
Agreement between generic and type-specific detection in cervical and urine samples taken from HIV-positive women.

Agreement between paired samples showed that generic viral detection had greater than 50% agreement in the results obtained for the three sets of primers used, while greater agreement was found for HPV-33 and HPV-45 types for type-specific identification (even though these were the least prevalent in the population being studied). However, the κ values gave poor correlation in all cases (Figure 3).

The urine sample's diagnostic performance revealed 68.8% sensitivity (60.5–76.2 95%CI), 50% specificity (36.8–63.2 95%CI), 76.7% positive predictive value (PPV) (68.5–83.7 95%CI), 40% negative predictive value (NPV) (28.9–52 95%CI) and a 0.59 ROC area (0.52–0.67 95%CI).

## Discussion

Developing cervical cancer has been related to factors determining its progression, including the type of HPV infection, viral load and persistence of the infection [35]. Nevertheless, as most women have an efficient immune system they can manage to rid themselves of infection within a period of less than two years [36].

However, when immune system activity becomes compromised, as in HIV-positive women, the elimination of concomitant infection is less efficient; a clear example lies in that described for women suffering simultaneous HIV-HPV infection whose natural history of infection becomes altered, thereby leading to the appearance of cervical lesions in less time. This is related to a reduction in HPV elimination rates, greater efficiency regarding the cellular transformation of all viral types and lower lesion regression rates [4].

According to the data obtained in this study, DNA integrity confirmed by amplifying two segments of the human β–globin gene (as an indirect measurement method) revealed that the percentage of samples having degraded or non-amplifiable DNA were low in both cervical and urine samples, thereby highlighting that the latter also represents a good source of DNA for amplifying specific targets using molecular biology techniques and could thus be considered as a useful cervical screening tool (in spite of 30% inhibition having been reported for such amplification) [37], [38].

The frequency of HPV infection detected in the present population agreed with that reported in previous studies carried out on populations having similar characteristics, such as that reported by Ferenczy *et al*., who described 73.6% crude HPV infection prevalence from cervical samples taken from sexually-active HIV-positive women [3]. Nevertheless, HPV infection prevalence in urine in the present study was lower than that in cervical samples; similar data have been reported previously for this type of sample [39]. Such difference in viral detection percentage could have been related to the low number of exfoliated cervical cells present in urine, to the presence of PCR inhibitors in this sample [37] or to methodological issues related with sampling strategies, storage conditions, sample manipulation and DNA extraction method that could affect the HPV-DNA detection [15]; therefore is necessary to continue working on the improvement of protocols for HPV-DNA detection from urine sample.

Regarding type-specific distribution, the data obtained from cervical samples agreed with published reports concerning the general Colombian population, HPV-16 being the most prevalent type, followed by HPV-31 [18]. However, urine samples' type-specific distribution profile revealed some differences compared to that for the cervical samples, HPV-18 being the second most prevalent type, this being similar to worldwide data reported in the pertinent literature [40]. It was also found that HPV-58 and HPV-45 were the only two viral types more prevalent in urine samples than in cervical samples, which could have been related to the fact that some viral types may preferentially infect the vagina's keratinized tissue than the non-keratinized tissue of the cervix [41]; however, more research needs to be done into HPV infection profiles regarding different areas of the lower genital tract.

In addition, the variations in HPV type-specific distribution profile could have been related to the presence of HIV infection as it has been described that such distribution in immunologically-compromised women could vary; moreover, it has been described that such incidence is 16 times higher in the immunologically-compromised group than that found in immunologically-competent women [3]. An additional explanation for the different viral type distribution between samples could be attributable to a varying exfoliation pattern in cells infected with each viral type, however, it has not yet been established whether exfoliated cells in urine are influenced by viral infection type or the state of infection [15].

Coinfection was found in both urine and cervical samples in around half the study population; this could have been attributed to the low infection elimination rate allowing different viral types to settle in the cervical epithelium; multiple infection events could have been also due to the reduced systemic and local cell immunity found in HIV-positive women [12].

There was poor agreement between generic and type-specific identification results; this may have been related to the samples' different nature, as well as HPV tropism for cervical epithelium. A lower number of viral copies in urine are expected regarding cervical samples, as the latter would have been taken from the pathogen's direct localization site.

Interestingly, the test involving self-collected urine samples had greater sensitivity (68.8% in this study vs. 55.3%) and more specificity (50.0% in this study vs. 44.9%) for detecting HPV-DNA compared with a previous study using the same identification protocol [19], which could indicate a potential use for the clinical application of this sample source. Nevertheless, additional studies must be carried out in the general population for determining clinical applicability, storage conditions, suitable extraction method, the most appropriate urine fraction to be used in the molecular analysis, and other factors that could affect the diagnostic performance of this sample source.

Developing strategies in cervical cancer control and prevention programs will be particularly determinant in contributing towards increasing coverage, sample taking, adherence and follow-up of women, mainly those presenting some type of immunosuppression. According to the results obtained here, self-sampling methods, such as urine sampling, could be taken into account as useful tools for preventing this pathology, since they offer good diagnostic performance and greater acceptability among women.
